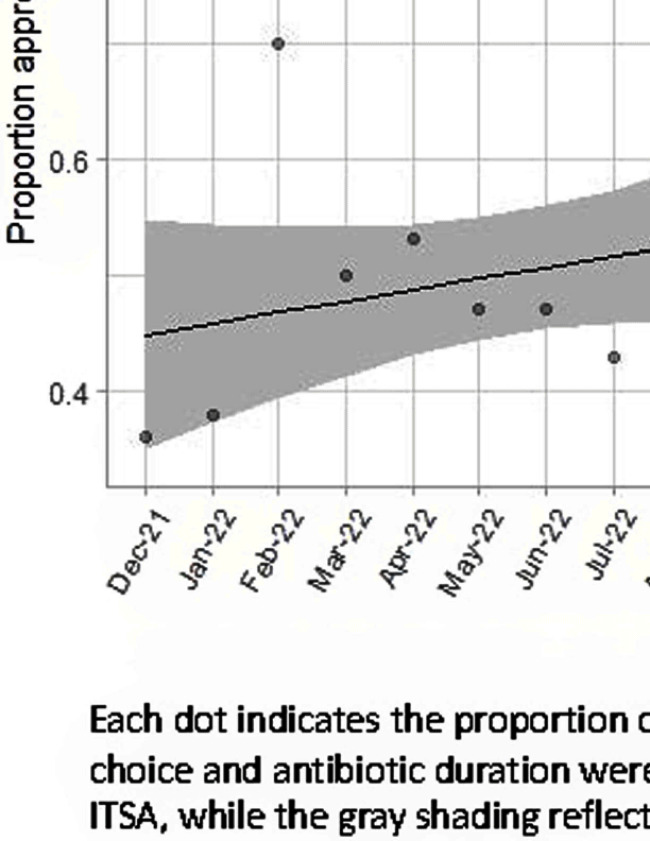# Improving antibiotic use for community acquired pneumonia in hospitalized children through electronic feedback reports

**DOI:** 10.1017/ash.2024.164

**Published:** 2024-09-16

**Authors:** Kathleen Chiotos, Lauren Dutcher, Robert Grundmeier, Didien Meyahnwi, Ebbing Lautenbach, Melinda Neuhauser, Keith Hamilton, Anne Jaskowiak, Leigh Cressman, Yun Li, Julie Szymczak, Brandi M. Muller, Jeffrey Gerber

**Affiliations:** Childrens Hospital of Philadelphia; University of Pennsylvania; Perelman School of Medicine, University of Pennsylvania; CDC DHQP; Hospital of the University of Pennsylvania; University of Pennsylvania Perelman School of Medicine; University of Pennsylvania/Dept. of Biostatistics, Epidemiology and Informatics; University of Utah School of Medicine; University of Utah; University of Pennsylvania School of Medicine

## Abstract

**Background:** Feedback reports summarizing clinician performance are effective tools to improve antibiotic stewardship in the ambulatory setting, but few studies have evaluated their effectiveness for pediatric inpatients. We developed and implemented feedback reports reflecting electronically-derived measures of appropriate antibiotic choice and duration for community acquired pneumonia (CAP) and measured their impact on appropriate antibiotic use in children hospitalized for CAP. **Methods:** We performed a single center quasi-experimental study including children 6 months to 17 years hospitalized for CAP between 12/1/2021-11/30/2023. Children with chronic medical conditions, ICU stays >48 hours, and outside transfers were excluded. The intervention occurred in 11/2022 and included clinician education, a monthly group-level feedback report disseminated by email (Figure 1), and a monthly review of clinician performance during a virtual quality improvement meeting. Patient characteristics were compared using chi-square or Wilcoxon rank sum tests. Interrupted time series analysis (ITSA) was used to measure the immediate change in the proportion of CAP encounters receiving both the appropriate antibiotic choice and duration, as well as the change in slope from the preintervention to the postintervention periods. Choice and duration were analyzed separately using ITSA as a secondary analysis. **Results:** There were 817 CAP encounters, including 420 preintervention and 397 postintervention. Patients admitted in the postintervention period were older (median age 2 years vs 3 years, P=0.03), but otherwise there were no differences in race, ethnicity, sex, ICU admission, or complicated pneumonia. Preintervention, 52% of encounters received both the appropriate antibiotic choice and duration; 96% of encounters received the appropriate antibiotic choice and 54% received the appropriate duration. The ITSA demonstrated an immediate 16% increase in the proportion of patients receiving both appropriate antibiotic choice and duration (95% confidence interval, 1-31%; P = 0.047) and no significant further increase over time following the intervention (P = 0.84) (Figure 2). When antibiotic choice was analyzed separately by ITSA, there was no immediate change or change over time in the proportion of patients receiving the appropriate antibiotic choice. In the ITSA of duration alone, there was an immediate 17% increase in the proportion receiving the appropriate duration (95% confidence interval, 2-33%; P = 0.03) and no change over time. **Conclusion:** Feedback reports generated from electronically-derived metrics of antibiotic choice and duration, combined with ongoing clinician education, increased the proportion of children with CAP treated with the appropriate antibiotic duration. Electronic feedback reports are a scalable and impactful intervention to improve antibiotic use in children hospitalized with CAP.

**Figure:**
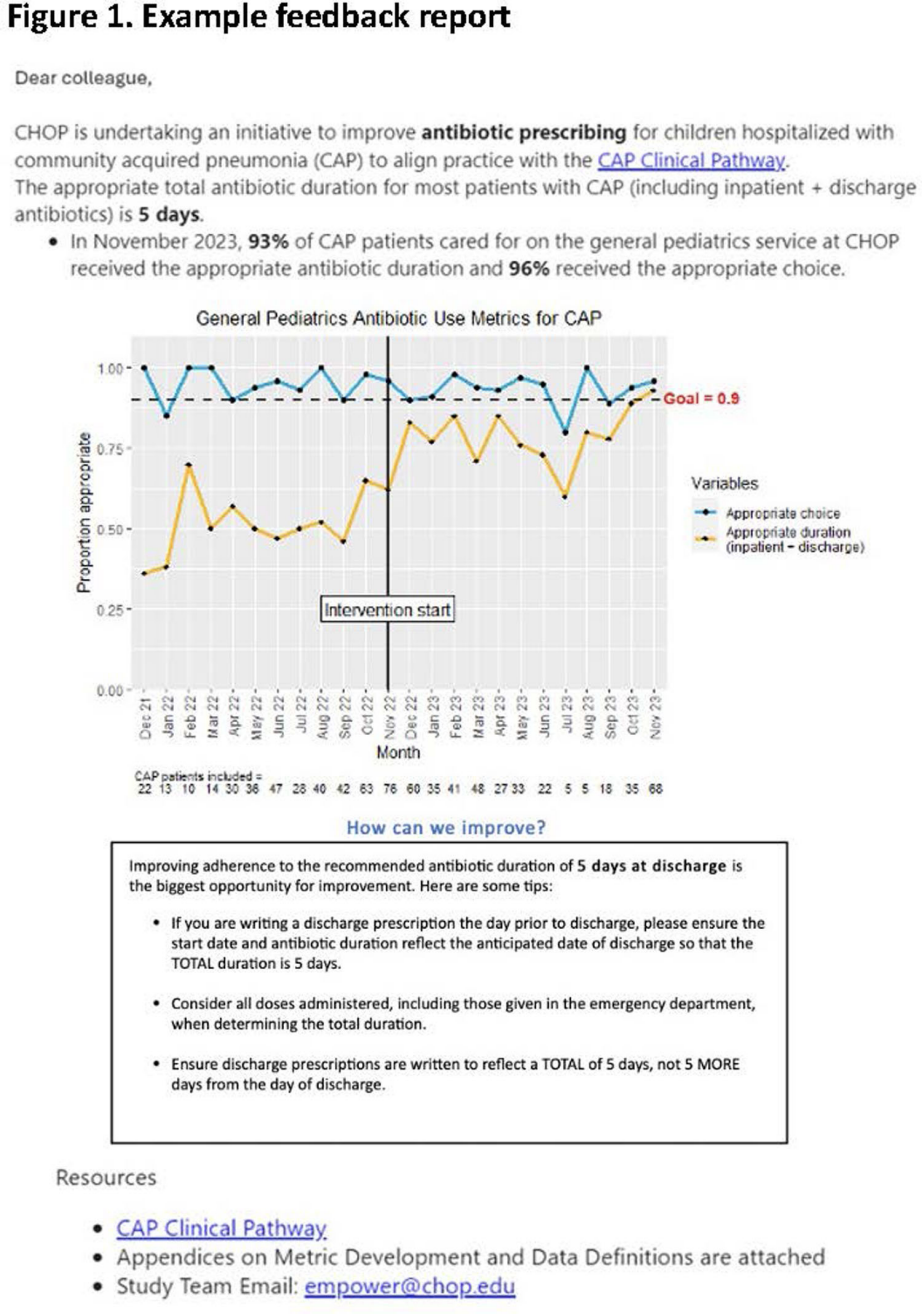

**Figure:**